# Association study of *FOXO3A*SNPs and aging phenotypes in Danish oldest-old individuals

**DOI:** 10.1111/acel.12295

**Published:** 2014-12-02

**Authors:** Mette Soerensen, Marianne Nygaard, Serena Dato, Tinna Stevnsner, Vilhelm A Bohr, Kaare Christensen, Lene Christiansen

**Affiliations:** 1 The Danish Aging Research Center, Epidemiology, Biostatistics and Biodemography, Institute of Public Health, University of Southern DenmarkJ.B. Winsloews Vej 9B, 5000, Odense C, Denmark; 2 Department of Clinical Genetics, Odense University HospitalSdr. Boulevard 29, 5000, Odense C, Denmark; 3 Department of Biology, Ecology and Earth Science, University of CalabriaPonte Pietro Bucci cubo 4C, 87036, Rende (Cs), Italy; 4 The Danish Aging Research Center, Department of Molecular Biology and Genetics, Aarhus UniversityC. F. Moellers Allé 3, 8000, Aarhus C, Denmark; 5 Laboratory of Molecular Gerontology, National Institute on Aging, National Institute of Health251 Bayview Boulevard, 21224-6825, Baltimore, MD, USA; 6 Department of Biochemistry and Pharmacology, Odense University HospitalSdr. Boulevard 29, 5000, Odense C, Denmark

**Keywords:** aging phenotypes, association study, Forkhead box O3, oldest-old, SNPs

## Abstract

*FOXO3A*variation has repeatedly been reported to associate with human longevity, yet only few studies have investigated whether *FOXO3A*variation also associates with aging-related traits. Here, we investigate the association of 15 *FOXO3A*tagging single nucleotide polymorphisms (SNPs) in 1088 oldest-old Danes (age 92–93) with 4 phenotypes known to predict their survival: cognitive function, hand grip strength, activity of daily living (ADL), and self-rated health. Based on previous studies in humans and foxo animal models, we also explore self-reported diabetes, cancer, cardiovascular disease, osteoporosis, and bone (femur/spine/hip/wrist) fracture. Gene-based testing revealed significant associations of *FOXO3A*variation with ADL (*P* = 0.044) and bone fracture (*P* = 0.006). The single-SNP statistics behind the gene-based analysis indicated increased ADL (decreased disability) and reduced bone fracture risk for carriers of the minor alleles of 8 and 10 SNPs, respectively. These positive directions of effects are in agreement with the positive effects on longevity previously reported for these SNPs. However, when correcting for the test of 9 phenotypes by Bonferroni correction, bone fracture showed borderline significance (*P* = 0.054), while ADL did not (*P* = 0.396). Although the single-SNP associations did not formally replicate in another study population of oldest-old Danes (*n* = 1279, age 94–100), the estimates were of similar direction of effect as observed in the Discovery sample. A pooled analysis of both study populations displayed similar or decreased sized *P*-values for most associations, hereby supporting the initial findings. Nevertheless, confirmation in additional study populations is needed.

## Introduction

Genetic factors contribute to the variation in human lifespan as well as to aging-related traits such as physical and cognitive function (Frederiksen *et al*., 2002; McGue & Christensen, 2002; Hjelmborg *et al*., 2006). The forkhead box O3 gene (*FOXO3A*) may, due to the verification in several study populations (Willcox *et al*., 2008; Anselmi *et al*., 2009; Flachsbart *et al*., 2009; Li *et al*., 2009; Pawlikowska *et al*., 2009; Soerensen *et al*., 2010; Zeng *et al*., 2010; Di Bona *et al*., 2013; Bao *et al*., 2014; Broer *et al*., 2014), be considered a second consistently confirmed longevity-associated gene in addition to the apolipoprotein E gene (*APOE)*. However, only few studies have investigated whether *FOXO3A*variation might also associate with aging-related phenotypes in the oldest-old.

*FOXO3A*is part of the insulin/insulin-like growth factor 1 signaling pathway, one of the key candidate pathways of longevity (Christensen *et al*., 2006). Together with *FOXO1A*,*FOXO4,*and *FOXO6*,*FOXO3A*constitutes the FoxO family of transcription factors, which regulates the expression of numerous downstream genes involved in a wide range of biological processes including apoptosis, cell cycle transition, DNA repair, oxidative stress, cell differentiation, and glucose metabolism (reviewed in (Huang & Tindall, 2007)).

Several animal studies have aimed to shed light on the physiological effects of loss-of-function or overexpression of foxo3a or its homologues. For example, lack of *foxo*in fruit flies (*Drosophila Melanogaster*) was, among other things, observed to increase the sensitivity to oxidative stress (Junger *et al*., 2003), while overexpression in the fat body was reported to reduce fecundity and increase lifespan in females (Giannakou *et al*., 2004). In zebra fish (*Danio rerio*), neural developmental defects were detected after knockdown of foxo3a, an effect likely mediated via increased apoptosis (Peng *et al*., 2010). In mice (*Mus musculus*) knockout of *Foxo3*has for instance been found to cause hematological abnormalities, decreased glucose uptake in glucose tolerance tests, and increased follicular activation, the latter leading to depletion of functional follicles, oocyte death, and infertility in female mice (Castrillon *et al*., 2003). In other mouse studies, *Foxo3*knockout induced incorrect immune responses, among others manifested as reduced numbers of B-cells (Hinman *et al*., 2009) and auto inflammation (Lin *et al*., 2004). Furthermore, *Foxo3*knockout has been reported to affect the maintenance of hematopoietic stem cells; for example, manifested as reduced autophagy after metabolic stress (Warr *et al*., 2013) and as reduced self-renewal in general, the latter likely impairing the maintenance of the stem cells during aging (Miyamoto *et al*., 2007). Tissue-specific mouse models have also been made, for instance cardiac-specific constitutive expression of *foxo3*was, among others, described to result in decreased heart weight (Schips *et al*., 2011), while constitutive expression in rat substantia nigra led to neuronal loss (Pino *et al*., 2014). Finally, as foxo1, foxo3, and foxo4 have overlapping functions, conditional triple knockout mice have been generated (reviewed in (Arden, 2008)). These mice were, among other things, reported to have decreased insulin levels in liver-specific knockouts (Haeusler *et al*., 2010), increased numbers of thymic lymphomas and hemangiomas (Paik *et al*., 2007), and reduced bone formation and bone mass (Ambrogini *et al*., 2010). In the latter study, osteoblast-specific overexpression of *foxo3*was found to increase bone formation (Ambrogini *et al*., 2010).

A few studies in humans have explored the association between *FOXO3A*variation and aging-related phenotypes in the oldest-old. Kuningas *et al*. (2007) observed an increased risk of stroke, but not of diabetes or cardiovascular disease (CVD), in elderly carriers of two specific *FOXO3A*haplotypes. A borderline significant association was seen for cardiovascular mortality risk, yet not for cancer mortality risk. Willcox *et al*. (2008) found no significant association of rs2802292 with stroke, diabetes, coronary heart disease, or cancer when long-lived individuals were investigated separately. However, when average-lived individuals from the same birth cohorts were included, the single nucleotide polymorphism (SNP) displayed association with coronary heart disease and health status. Also, a borderline significant association with cancer was noted. Pawlikowska *et al*. (2009) examined rs1935949 and rs4946935 in long-lived individuals and participants of their prospective cohort, who died from specific causes. rs4946935 showed association with death caused by cancer, while both SNPs showed association with death caused by atherosclerosis. Although a replication sample did not reveal significance, meta-analyses did support the associations.

To further explore whether *FOXO3A*variation associates with aging-related traits likely influencing longevity, we investigated the association of 15 *FOXO3A*tagging SNPs with phenotypes previously shown to predict survival in the oldest-old studied here (Nybo *et al*., 2003): cognitive function, physical function (as measured by hand grip strength), activity of daily living (ADL), and self-rated health. In addition, we also explored the prevalence of self-reported diabetes, cancer, CVD, osteoporosis, and bone (femur/spine/hip/wrist) fracture (bone fracture).

## Results

**Figure 1:**
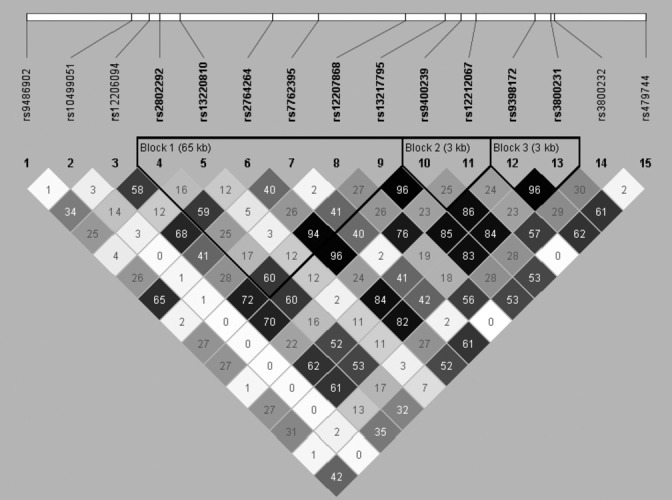
Linkage disequilibrium plot of the 15 *FOXO3A*SNPs under study. Notes: The values are R^2^ (a value of 100 reflects complete dependency between SNPs), and the colors reflect R^2^ (the darker color the higher the R^2^). kb: kilobases.

The characteristics of the study populations are shown in Table S1 (Supporting information), while the 15 *FOXO3A*SNPs are given in Fig.1.

### Gene-based association study in the Discovery sample

To investigate the overall association of *FOXO3A*variation with the nine phenotypes, a gene-based analysis was performed. The data are summarized in Table1. Only ADL and self-reported bone fracture displayed p-gene values below 0.05: p-gene (ADL) = 0.044 and p-gene (bone fracture) = 0.006. These p-gene values are corrected for the test of 15 SNPs, but not for the test of nine phenotypes. When conducting a strict Bonferroni correction, the association of *FOXO3A*variation with bone fracture was borderline significant (p-gene = 0.054), while the association of *FOXO3A*variation with ADL was not (p-gene = 0.396).

**Table 1:** Gene-based association of *FOXO3A*variation and the nine phenotypes in the Discovery sample

Phenotype	*n*	p-gene
Cognitive composite score	1026	0.851
Self-reported CVD	1086	0.777
Hand grip strength	980	0.281
Self-reported diabetes	1087	0.228
Self-reported cancer	1085	0.226
Self-reported osteoporosis	1061	0.221
Self-rated health	1040	0.163
Activity of daily living	1086	**0.044**
Self-reported bone fracture	1063	**0.006**

*n*, number of individuals with data (in total 1088 individuals were genotyped); p-gene, set-based value obtained after 10000 permutations; CVD, cardiovascular disease. p-gene values < 0.05 are shown in bold.

### Effects at the single-SNP level for ADL and bone fracture

The gene-based analysis gives no overall effect estimate. Hence, the direction of effect of the associations observed at the gene-level must be represented by the size and direction of effect of the coefficients of the single-SNP tests, which were the basis of the gene-based analysis. These data are summarized in Table2. Five of the 15 SNPs showed p-SNP values below 0.05 for ADL, while 3 SNPs showed nonsignificant borderline association (p-SNPs: 0.055–0.074). For the minor alleles of all of these SNPs the direction of effect on ADL was positive, corresponding to a decreased disability (see Table2A). With respect to bone fracture, 10 of the 15 SNPs showed *P*-values below 0.05. Again, the direction of effect was positive for all minor alleles of these SNPs, corresponding to a decreased risk of bone fracture (see Table2B).

**Table 2:** Single-SNP analysis in the Discovery sample of *FOXO3A*variation and (A) activity of daily living and (B) bone (femur/spine/hip/wrist) fracture

(A) Activity of daily living (*n* = 1086)				(B) Bone fracture (*n* = 1063)			
SNP	β-coef.	p-SNP	95% CI	SNP	OR	p-SNP	95% CI
rs9400239	0.07	**0.020**	0.01; 0.14	rs9486902	0.60	**0.001**	0.45; 0.81
rs9398172	0.07	**0.021**	0.01; 0.14	rs7762395	0.57	**0.001**	0.42; 0.78
rs13217795	0.07	**0.024**	0.01; 0.14	rs479744	0.69	**0.007**	0.52; 0.90
rs2802292	0.07	**0.026**	0.01; 0.13	rs3800231	0.72	**0.008**	0.57; 0.92
rs2764264	0.07	**0.042**	0.01; 0.13	rs9398172	0.74	**0.012**	0.58; 0.94
rs479744	0.07	***0.055***	−0.01; 0.14	rs9400239	0.75	**0.017**	0.59; 0.95
rs3800231	0.06	***0.063***	−0.01; 0.12	rs2764264	0.75	**0.020**	0.59; 0.96
rs7762395	0.07	***0.074***	−0.01; 0.15	rs13217795	0.76	**0.028**	0.60; 0.97
rs10499051	0.08	0.133	−0.02; 0.18	rs12206094	0.77	**0.035**	0.61; 0.98
rs12206094	0.05	0.149	−0.02; 0.11	rs2802292	0.80	**0.049**	0.64; 0.99
rs9486902	0.05	0.171	−0.02; 0.13	rs13220810	0.88	0.303	0.70; 1.12
rs3800232	0.04	0.392	−0.05; 0.13	rs12207868	1.17	0.362	0.83; 1.65
rs12207868	0.01	0.828	−0.09; 0.11	rs10499051	1.12	0.549	0.78; 1.61
rs12212067	0.01	0.869	−0.09; 0.10	rs3800232	0.94	0.711	0.68; 1.30
rs13220810	-0.01	0.905	−0.07; 0.06	rs12212067	1.03	0.874	0.73; 1.45

The single-SNP tests were the basis of the gene-set analysis in the Discovery sample. *n*, number of individuals with data; β-coef., beta coefficient; OR, odds ratio; CI, confidence interval; p-SNP values < 0.05 are shown in bold, while p-SNP values > 0.05 < 0.10 are shown in bold and italics.

### Replication study of the ADL and bone fracture findings

Five SNPs were investigated in the Replication sample: rs12206094, rs2802292, rs3800231, rs479744, and rs7762395 (see Experimental procedures). None of the initial findings could be formally replicated. However, all of the effect estimates observed in the Replication sample were in the same direction, although generally attenuated, as compared to the Discovery sample (compare Table3A to B, and Table3D to E).

**Table 3:** Replication study of activity of daily living and bone fracture findings

Activity of daily living									
	(A) ADL in Discovery sample (*n* = 1086)			(B) ADL in Replication sample (*n* = 1279)			(C) ADL Pooled analysis		
SNP	β-coef.	p-SNP	95% CI	β-coef.	p-SNP	95% CI	β-coef.	p-SNP	95% CI
rs2802292	0.07	0.026	0.01; 0.13	0.04	0.183	−0.02; 0.09	0.05	0.017^*^	0.01; 0.09
rs479744	0.07	0.055	−0.01; 0.14	0.02	0.582	−0.05; 0.09	0.04	0.112	−0.01; 0.09
rs3800231	0.06	0.063	−0.01; 0.12	0.03	0.331	−0.03; 0.09	0.04	0.054^*^	−0.01; 0.09
rs7762395	0.07	0.074	−0.01; 0.15	0.03	0.514	−0.05; 0.10	0.04	0.114	−0.01: 0.10
rs12206094	0.05	0.149	−0.02; 0.11	0.04	0.202	−0.02; 0.10	0.04	0.069^*^	−0.01; 0.08

Bone fracture									
	(D) BF in Discovery sample (*n* = 1063)			(E) BF in replication sample (*n* = 691)			(F) BF Pooled analysis		
SNP	OR	p-SNP	95% CI	OR	p-SNP	95% CI	OR	p-SNP	95% CI
rs7762395	0.57	0.001	0.42; 0.78	0.90	0.542	0.64; 1.26	0.71	0.003	0.56; 0.89
rs479744	0.69	0.007	0.52; 0.90	0.86	0.311	0.63; 1.16	0.76	0.006^*^	0.62; 0.92
rs3800231	0.72	0.008	0.57; 0.92	0.88	0.361	0.68; 1.15	0.80	0.012	0.67; 0.95
rs12206094	0.77	0.035	0.61; 0.98	0.97	0.817	0.73; 1.28	0.85	0.085	0.71; 1.02
rs2802292	0.80	0.049	0.64; 0.99	0.87	0.287	0.67; 1.12	0.83	0.033^*^	0.70; 0.99

ADL, activity of daily living; *n*, number of individuals with data; β-coef., beta coefficient; CI, confidence interval; BF, bone fracture; OR, odds ratio;

* SNPs for which the pooled analysis showed lower p-SNPs as compared to the analysis of the Discovery sample alone.

In a pooled analysis of the data from both study populations, all five SNPs showed nominal or borderline significant (p-SNP>0.05 < 0.10) association with bone fracture, and the significance was increased for rs2802292 and rs479744 as compared to the Discovery sample (compare Table3D to F). With respect to ADL, rs12206094, rs2802292, and rs3800231 showed nominal or borderline significant association in the pooled analysis and furthermore showed lower sized p-SNP values as compared to the Discovery sample (compare Table3A to C). The effect estimates of the pooled analysis were of the same direction, as compared to the analyses in the Discovery sample alone and in the Replication sample alone.

## Discussion

In this study, we investigated the association of variation in the second consistently confirmed longevity-associated gene, *FOXO3A*, with aging-related phenotypes in the oldest-old. In the Discovery sample, we explored four phenotypes known to predict their survival, that is, cognitive function, self-reported health, hand grip strength, and ADL. Moreover, we included data on five self-reported diseases: diabetes, cancer, CVD, osteoporosis, and bone fracture, as these are either investigated in genetic association studies and/or are supported by foxo animal models.

We found *FOXO3A*variation to show nominal significant association with two of the investigated phenotypes; ADL and bone fracture (see Table1). This does, however, not exclude relevance of *FOXO3A*variation for the remaining seven phenotypes or for the physiological processes behind these phenotypes; it is possible that more statistical power is needed to detect such associations, especially if these are of small effect size (see Experimental procedures). Furthermore, as the foxo3a protein has a wide array of downstream targets, which themselves affect a wide range of cellular and physiological processes, it may simply be difficult to pinpoint the candidate phenotypes, which *FOXO3A*potentially affects.

Contrary to our results, two previous studies (Willcox *et al*., 2008; Pawlikowska *et al*., 2009) described association of single *FOXO3A*SNPs with cancer and health status, respectively. In a retrospective lookup, none of these associations could, however, be replicated in our data (data not shown). The lack of concordance could be due to differences in phenotypes or study designs, for example the present study investigates oldest-old only, while early dying controls were included in the other studies. Hence, it might be speculated that *FOXO3A*influences health status and cancer prevalence only at younger ages. That associations can change with age has been described previously (Jacobsen *et al*., 2010). Similarly, due to the study by Kuningas *et al*. (2007), we performed a retrospective analysis of CVD data restricting to stroke and found no association (data not shown). This is, however, in accordance with the previous study, as they only found haplotype specific and not single variant specific associations (Kuningas *et al*., 2007). A few additional studies have indicated association of *FOXO3A*variation with insulin sensitivity, for example fasting insulin levels, in young and/or elderly individuals (Willcox *et al*., 2008; and Banasik *et al*., 2011). As we did not have data on insulin sensitivity, we could not test this. However, we did not find any association with self-reported diabetes. One explanation could be that such an association is most relevant at younger ages and not among the oldest-old.

The single-SNP statistics behind the gene-based analyses of ADL and bone fracture indicated positive effects of the minor alleles of the majority of the 15 *FOXO3A*SNPs; that is, increased ADL (decreased disability) and reduced risk of bone fracture. The associations were, however, not statistically significant for all SNPs (see Tables2A,B). Further supporting an effect of variation in *FOXO3A*on ADL and bone fracture, it can be noticed that the 15 SNPs were distributed over the entire gene region and that, at least for some of the SNPs, there was rather modest linkage disequilibrium (LD) (see Fig.1). Hence, it seems that the estimates, obtained for either ADL or bone fracture, are not completely redundant. Furthermore, it might also suggest that the findings are not chance findings.

Our findings are strengthened by the fact that for all of the 8 SNPs suggested to associate with ADL and the 10 SNPs associated with bone fracture, the minor alleles (or the minor alleles of their proxies (R^2^ > 0.8)) have previously been reported to pose a positive effect on human longevity in between 1 and 5 studies (Willcox *et al*., 2008; Anselmi *et al*., 2009; Flachsbart *et al*., 2009; Li *et al*., 2009; Pawlikowska *et al*., 2009; Soerensen *et al*., 2010; Zeng *et al*., 2010; Broer *et al*., 2014). This convincing overlap in SNP associations could indicate an influence of *FOXO3A*variation directly on both the aging phenotypes and on longevity or it might alternatively suggest that an effect on the aging phenotypes could affect longevity.

Interestingly, 8 of the 15 *FOXO3A*SNPs showed borderline or nominal significant association with both ADL and bone fracture, and the coefficients for both phenotypes showed the same direction of effect. In retrospective regression analyses of ADL and each of these SNPs additionally adjusting for bone fracture, significance was observed between ADL and bone fracture, and the associations between ADL and the SNPs were slightly attenuated (data not shown). The SNP associations were, however, still found to be significant or borderline significant (data not shown). The same was found when additionally adjusting the regression analyses of bone fracture and each SNP for ADL (data not shown). The associations observed between ADL and bone fracture imply that the associations between the *FOXO3A*SNPs and the two phenotypes could partly be due to shared underlying factors, while the preservation of (borderline) significance for the SNPs implies that the associations with the two phenotypes must also be explained by nonshared underlying factors. Considering that *FOXO3A*affects a wide range of target genes, which in turn influence various biological processes, the potential shared and nonshared factors are numerous.

The association between *FOXO3A*variation and bone fracture was not accompanied by a concurrent association with osteoporosis. However, these two phenotypes cannot be expected to be completely interchangeable, as osteoporosis is often underdiagnosed and undertreated in Denmark (Eiken & Abrahamsen, 2010). This aspect appears to be reflected in the prevalence of bone fracture and osteoporosis in the Discovery sample; despite some concordance between the two phenotypes (*P*-value (χ2 test) < 0.001), only 9.2% of the individuals reported an osteoporosis diagnosis, while 25% reported a bone fracture. Furthermore, due to the nature of the survey data, we cannot affirm that all fractures are osteoporotic; for this end, information on high- or low-energy fractures and the age at the time of fracture would be needed, that is, low-energy fractures occurring at higher ages would be more likely to be osteoporotic. Nevertheless, when retrospectively inspecting the coefficients of the single-SNP analysis of osteoporosis, 9 of the 10 SNPs, which showed a nominal significant positive effect on bone fracture, also indicated a positive effect on osteoporosis, although it was not statistically significant (data not shown).

We could not formally replicate the associations of *FOXO3A*variation with ADL and bone fracture in another sample of Danish oldest-olds. There are a number of possible reasons for this. First of all, it may indicate that these were merely chance findings or that the Replication sample could be underpowered with respect to small effect sizes. However, another explanation could be that the individuals of the Replication sample were slightly older (94.7–100.9 years) than the individuals of the Discovery sample (92.2–93.8 years). This could potentially be of importance if the associations are not constant with age. As mentioned earlier, such changes in association with age have been reported previously (Jacobsen *et al*., 2010). Still, the Replication sample was the best obtainable study population, as the Replication sample was surveyed using the exact same questionnaire and assessment instrument as the Discovery sample. Finally, the effect estimates are of critical importance (Olsen *et al*., 2010) and for the majority of the SNPs, the effect estimates [β-coefficients and odds ratios (ORs)] of the Replication sample had similar direction as those observed in the Discovery sample (Table3). In the pooled analysis (Table3), this was further supported by comparable direction of the effect estimates and by a lowering of p-SNP values for some of the SNPs. In general, we believe that these findings lend suggestive support to the associations seen in the Discovery sample, although additional studies are needed to enable conclusion.

In this study, we for the first time suggest the association of variation in the second consistently confirmed longevity-associated gene, *FOXO3A*, with ADL and bone fracture. Such effects could mediate the influence of *FOXO3A*variation on longevity; however, further explorations in additional study populations are needed before conclusions can be drawn.

## Experimental procedures

### Subjects

The Discovery sample was 1200 participants randomly drawn from 1651 eligible members of the Danish 1905 Birth Cohort Study (Nybo *et al*., 2001), a nationwide survey of an entire birth cohort. The *FOXO3A*genotype data set is described in (Soerensen *et al*., 2010, 2012). After data cleaning, data were available for 1088 individuals with an age range of 92.2–93.8 years (mean: 93.1 years) and a gender distribution of 29% males and 71% females.

For replication of the findings observed in the Discovery sample, we used data on 1279 individuals from two additional population-based surveys of oldest-old Danes: the Danish 1910 and 1915 Birth Cohort Studies (unpublished data and (Christensen *et al*., 2013)). These surveys were conducted similar to the Danish 1905 Birth Cohort Study. The age range was 94.7–100.9 years (mean: 96.0 years), and the gender distribution was 27% males and 73% females.

Permission to collect blood samples and to use survey information was granted by The Danish Regional Committees on Biomedical Research Ethics.

### Genotype data

The 15 *FOXO3A*SNPs investigated in the Discovery sample are located in position 108982719–109113664 of chromosome 6, which corresponds to *FOXO3A*and 5000 base pairs (bp) upstream and 1000 bp downstream (NCBI assemble 36). Details on how these *FOXO3A*SNPs were chosen and the generation of Illumina GoldenGate (Illumina Inc., San Diego, CA, USA) genotype data were previously described (Soerensen *et al*., 2010, 2012). The LD plot in Fig.1 is based on the genotype data of the Discovery sample, and the plot was generated in the Haploview software (http://www.broadinstitute.org/scientific-community/science/programs/medical-and-population-genetics/haploview/haploview, (Barrett *et al*., 2005)).

To perform a replication study of the initial findings, the LD pattern of the 10 SNPs, which showed significance in the Discovery sample, was investigated in the SNAP Proxy database (http://www.broadinstitute.org/mpg/snap/ldsearch.php). The r2 > 0.8 criterion was used with data from the 1000 Genomes project (Abecasis *et al*., 2010). Consequently, five SNPs, rs12206094, rs2802292, rs3800231, rs479744 and rs7762395, were selected, which in general cover the same genetic variation as the 10 SNPs found to be significant in the Discovery sample. The DNA of the Replication sample was purified from blood spot cards using the Extract-N-AmpTM Blood PCR Kit (Sigma Aldrich, St. Louis, MO, USA), and genotyping was performed by standard TaqMan procedures, using predesigned TaqMan® SNP Genotyping Assays (Applied Biosystems, Foster City, CA, USA).

### Phenotype data

The phenotype data for all surveys were collected as part of a comprehensive home-based interview focusing on health and disease issues. Cognitive function was assessed by a cognitive composite score, which investigates verbal fluency, forward and backward digit span, and immediate and delayed recall (McGue & Christensen, 2002). Handgrip strength was determined using a handheld dynamometer (SMEDLEY'S dynamometer, Scandidact, Kvistgaard, Denmark); the maximum of three measurements with the strongest hand was used. Self-rated health was assessed by the question ‘How do you consider your health in general?’ with five response categories: very poor, poor, acceptable, good, and excellent. ADL was inspected by a five-item ADL disability score (based on the Katz ADL index (Katz *et al*., 1970)) about bathing, dressing, toileting, transfer and feeding; the score was categorized into ‘disabled = could do maximum two items’, ‘moderately disabled = could do three or four items, ‘ or ‘not disabled = could do all five items. ‘ Self-reported status on cancer, osteoporosis, and diabetes was evaluated by asking: ‘Did a doctor ever tell you that you have/had any of the following diseases? ‘ with the response categories ‘has now or has had‘ and ‘no. ‘ Self-reported CVD was obtained in the same way, yet with six diseases combined in one group; stroke, heart attack, angina pectoris, irregular heart rhythm, other heart problems, and treatment for hypertension with prescription medicine/drop. Self-reported fracture of the femur, spine, hip, and/or wrist was investigated, as these types of fractures are considered typical osteoporotic frailty fractures and likely indicate low bone density (Nguyen *et al*., 2001; Bathum *et al*., 2004; Broderick *et al*., 2013). Status on bone fracture was evaluated by asking ‘Did you fracture a bone after the age of 40? ‘, and if the answer was ‘yes‘ subsequently asking: ‘Where did you have the fracture? ‘ with five response categories ‘femur, ‘spine (also collapse), ‘hip, ‘wrist, ‘ and ‘other places. ‘ The individuals, whom reported at least 1 fracture of the femur, spine, hip, and/or wrist after age 40, were compared to the rest of the study population.

The phenotype data used for the Replication sample were based on data from the respective surveys, and the questions were identical to the questions in the Danish 1905 Birth Cohort Study.

### Statistical analyses

Set(gene)-based analyses of the 15 *FOXO3A*SNPs and each of the nine phenotypes were performed in Plink (Purcell *et al*., 2007); applying the most inclusive settings, that is set-max 99999, set-r2 1, and set-p 1. In the set-based test, linear regression was applied for analysis of the cognitive composite score, hand grip strength, ADL, and self-rated health, while logistic regression was used for analysis of status on cancer, diabetes, CVD, osteoporosis, and bone fracture. All set-based analyses were adjusted for age and sex, while the set-based analysis of the cognitive composite score was additionally adjusted for level of education (<7th grade, 7th–8th grade, 9th–10th grade or >11th grade), the set-based analysis of self-reported cancer was additionally adjusted for smoking status (current, former, or never smoker), the set-based analyses of hand grip strength and self-reported diabetes were additionally adjusted for body mass index (bmi), the set-based analysis of self-reported CVD was additionally adjusted for bmi and smoking status, and the set-based analyses of self-reported bone fracture and osteoporosis were additionally adjusted for bmi, smoking status, and hormone use after menopause (hormone therapy or no hormone therapy). In the set-based analysis, the single-SNP *P*-values are summarized by their overall mean, while considering LD; hence, the analysis estimates the average association of the *FOXO3A*SNPs with a given phenotype. The significance (p-gene) was determined by repeating this for 10000 permutations of the phenotype, consequently correcting for the test of 15 SNPs.

Analysis of the genotype and phenotype data from the Replication sample was performed by single-SNP analysis using the same single-SNP statistics as described above as part of the set-based test conducted for the Discovery sample. The analysis of bone fracture was restricted to the 691 individuals of the Replication sample for whom data on bmi were available. All replication analyses were done in the STATA 11.0 software (Stata Corporation, College Station, TX, USA).

### Power calculations

The set(gene)-based method applied in the present study is a power-full approach as it tests a group of SNPs in a gene and hereby increases power as compared to testing the individual SNPs separately. However, power calculations for set(gene)-based approaches are unfortunately not yet available. To inspect the effect sizes obtainable with good certainty (power>0.8) in the single-SNP analysis in the Discovery sample, we performed power calculations in the Quanto software (version Quanto1_2_4a, http://biostats.usc.edu/cgi-bin/DownloadQuanto.pl) assuming an additive model. For SNPs with a minor allele frequency (MAF) of 0.4, ORs of moderate size (OR < 0.60–0.78) should be obtainable for the binary phenotypes, while for a MAF of 0.1 sizeable ORs (ORs < 0.36–0.66) should be attainable. For the cognitive composite score, hand grip strength, ADL, and self-rated health, rather small beta-coefficients should be obtainable for a MAF of 0.4: >0.43, >0.86, >0.09 and >0.12, respectively (≈0.13 of a standard deviation for all four phenotypes). The same was true for the less frequent variants (MAF 0.1), where the values were >0.70, >1.42, >0.14, and >0.19, respectively (≈0.21of a standard deviation for all four phenotypes).

## Supporting Information

**Table S1.** Characteristics of the Discovery and Replication samples with respect to the phenotypes investigated.
