# Supplementary material for: Association study of FOXO3ASNPs and aging phenotypes in Danish oldest-old individuals
Source: Aging Cell. 2014 Dec 2;14(1):60–6. doi: 10.1111/acel.12295 (PMC4326903; doi:10.1111/acel.12295)
Supplement: Supplementary file 1 [file acel0014-0060-sd1.docx]

| **Discovery sample (N = 1,088)** | | |
| --- | --- | --- |
| **Phenotype** | **n** |  |
| Cognitive composite score (mean (SE)) | 1,039 | 0.32 (0.11) |
| Hand grip strength (mean (SE)) | 997 | 16.44 (0.21) |
| Activity of daily living (mean (SE))  Disabled/moderately disabled/not disabled  Disabled (%)/moderately disabled (%)/not disabled (%) | 1,086 | 2.41 (0.02)  117/403/566  10.8/37.1/52.1 |
| Self-rated health (mean (SE))  Very poor/poor/acceptable/good/excellent  Very poor (%)/poor (%)/acceptable (%)/good (%)/excellent (%) | 1,046 | 3.64 (0.03)  17/78/350/417/184  1.6/7.5/33.4/39.9/17.6 |
| Self-reported diabetes (disease Yes (%)) | 1,087 | 77 (7.1) |
| Self-reported cancer (disease Yes (%)) | 1,085 | 129 (11.9) |
| Self-reported cardiovascular disease (disease Yes (%)) | 1,086 | 544 (50.1) |
| Self-reported osteoporosis (disease Yes (%)) | 1,084 | 100 (9.2) |
| Self-reported bone fracture (disease Yes (%)) | 1,083 | 271 (25.0) |
| **Replication sample (N = 1,279)** | | |
| **Phenotype** | **n** |  |
| Activity of daily living (mean (SE))  Disabled/moderately disabled/not disabled  Disabled (%)/moderately disabled (%)/not disabled (%) | 1,274 | 2.36 (0.02)  164/492/618  12.9/38.6/48.5 |
| Self-reported bone fracture (disease Yes (%)) | 1,279 | 408 (31.9) |

**Supplementary Table 1:** Characteristics of the Discovery and Replication samples with respect to the phenotypes investigated.

Notes: N: number of individuals genotyped, n: number of individuals with phenotype data, SE: standard error
